# The effect of vitamin D supplementation and nutritional intake on skeletal maturity and bone health in socio-economically deprived children

**DOI:** 10.1007/s00394-021-02511-5

**Published:** 2021-02-20

**Authors:** Suma Uday, Semira Manaseki-Holland, Jessica Bowie, Mohamed Zulf Mughal, Francesca Crowe, Wolfgang Högler

**Affiliations:** 1 Department of Endocrinology and Diabetes, Birmingham Women’s and Children’s Hospital, Steelhouse lane, Birmingham, UK; 2 Institute of Metabolism and Systems Research, University of Birmingham, Edgbaston, Birmingham, UK; 3 Institute of Applied Health Research, University of Birmingham, Birmingham, UK; 4 College of Medical and Dental Sciences, University of Birmingham, Rm G31, Public Health Building, Edgbaston, Birmingham, B15 2TT UK; 5 Department of Paediatric Endocrinology, Royal Manchester Children’s Hospital, Manchester, UK; 6 Department of Paediatrics and Adolescent Medicine, Johannes Kepler University, Linz, Austria

**Keywords:** Malnutrition, Radiographs, Short stature, Vitamin D, Growth, Bone health

## Abstract

**Purpose:**

1. To determine the effect of vitamin D supplementation on bone age (BA), a marker of skeletal maturity, and Bone Health Index (BHI), a surrogate marker of bone density. 2. To characterise the differences in nutritional intake and anthropometry between children with advanced vs. delayed BA.

**Methods:**

The current study is a post hoc analysis of radiographs obtained as part of a randomised controlled trial. In this double-blind, placebo-controlled trial, deprived Afghan children (*n* = 3046) aged 1–11 months were randomised to receive six doses of oral placebo or vitamin D3 (100,000 IU) every 3 months for 18 months. Dietary intake was assessed through semi-quantitative food frequency questionnaires at two time points. Anthropometric measurements were undertaken at baseline and 18 months. Serum 25OHD was measured at five time points on a random subset of 632 children. Knee and wrist radiographs were obtained from a random subset (*n* = 641), of which 565 wrist radiographs were digitised for post-hoc analysis of BA and BHI using BoneXpert version 3.1.

**Results:**

Nearly 93% (522, male = 291) of the images were analysable. The placebo (*n* = 258) and vitamin D (*n* = 264) groups were comparable at baseline. The mean (± SD) age of the cohort was 2 (± 0.3) years. At study completion, there was no difference in mean 25-hydroxy vitamin D concentrations [47 (95% CI 41, 56) vs. 55 (95% CI 45, 57) nmol/L, *p* = 0.2], mean (± SD) BA SDS [− 1.04 (1.36) vs. − 1.14 (1.26) years, *p* = 0.3] or mean (± SD) BHI SDS [− 0.30 (0.86) vs. − 0.31 (0.80), *p* = 0.8] between the placebo and vitamin D groups, respectively. Children with advanced skeletal maturity (BA SDS ≥ 0) when compared to children with delayed skeletal maturity (BA SDS < 0), had consumed more calories [mean (± SD) calories 805 (± 346) vs 723 (± 327) kcal/day, respectively, p < 0.05], were significantly less stunted (height SDS − 1.43 vs. − 2.32, *p* < 0.001) and underweight (weight SDS − 0.82 vs. − 1.45, *p* < 0.001), with greater growth velocity (11.57 vs 10.47 cm/ year, *p* < 0.05).

**Conclusion:**

Deprived children have significant delay in skeletal maturation but no substantial impairment in bone health as assessed by BHI. BA delay was influenced by total calorie intake, but not bolus vitamin D supplementation.

**Supplementary Information:**

The online version contains supplementary material available at 10.1007/s00394-021-02511-5.

## Background

Severe malnutrition or undernutrition resulting in stunting, wasting and underweight is a major concern in children under 5 years in low- and middle-income countries [1]. Stunting (low height-for-age) reflects a failure to reach linear growth potential due to suboptimal health and/or nutritional conditions [2]. Wasting (low weight-for-height) mostly indicates a recent weight loss from acute starvation or severe disease process but can also indicate chronic unfavourable conditions [2]. An underweight (low weight-for-age) child can be wasted or stunted or both and underweight can, therefore, be a useful marker of long-term health and nutrition in the absence of wasting [2].

Afghanistan, has one of the world’s highest rates of undernutrition [3]. Stunting, wasting and underweight are present in 40.9%, 9.5%, and 25% of Afghan children aged under 5 years, respectively [4]. Vitamin D deficiency has been associated with stunted growth [5, 6] which is widely prevalent in Afghan children. In the 2013 National Nutrition Survey of Afghan children aged 6–59 months, nearly 17% had deficient [serum 25 hydroxy vitamin D (25OHD) < 20 nmol/L] and around 65% insufficient (20–50 nmol/L) vitamin D levels [4].

Nutritional status, including vitamin D status, not only influences growth but also skeletal maturity [7] in childhood. Skeletal maturity is best determined by assessing bone age (BA) on hand radiographs. Traditionally, BA has been manually assessed using the Greulich and Pyle [8] and the Tanner–Whitehouse [9] methods. More recently, automated BA assessment using sophisticated software such as BoneXpert (Visiana, Denmark) has been used. Such automated methods are standardised, less time consuming and minimise user dependence [10]. The BoneXpert software also measures a Bone Health Index (BHI) which is based on measurement of cortical thickness (T) in the middle three metacarpals. In fact, measuring T is one of the oldest methods of assessing bone density. Automated BHI in children has been shown to reflect cortical bone-mineral density assessed by densitometry [11, 12]. Reference data for automated BA and BHI exist for children aged > 5 years since 2009 [13]; however the reference data for children < 5 years have only very recently become available [14].

The positive effect of protein calorie dietary supplementation on the number of ossified centres (NOC’s) on hand radiographs, as a marker of skeletal maturity, has been described in one previous report [15]. However, the effect of vitamin D supplementation on BA and BHI in malnourished children has not been studied to date.

In addition, malnutrition in Afghan children is multifactorial involving nutritional and non-nutritional (limited access to health care, drinking water, poor sanitation) factors. Experts have disputed the primary cause of malnutrition with variable importance placed on low calorie intake [16]. We therefore, evaluated the nutritional and anthropometric differences in children with advanced vs delayed skeletal maturation.

## Aims

1. Evaluate the effect of quarterly bolus oral vitamin D supplementation on BA and BHI assessed by automated radiogrammetry.
2. Characterise the differences in nutritional intake and anthropometry between children with advanced vs delayed bone age.

## Methods

### Study design

The current study is a post-hoc analysis of radiographs obtained as part of a randomised controlled trial. A detailed description of the parent study methods has been published elsewhere [17]. Briefly, the study was a community-based, double-blind, randomised, placebo-controlled trial that was conducted between November 2007 and June 2009 in five deprived inner-city districts of Kabul, Afghanistan. The primary objective of the trial was to evaluate the effect of quarterly supplementation of 100,000 IU (2.5 mg) of vitamin D3 on the incidence and/or severity of childhood pneumonia.

### Approvals, consent and registration

The study protocol was approved by the Ethics and Review Board of the Ministry of Public Health of Afghanistan (Reference: 422328) and the Ethics Committee of the London School of Hygiene and Tropical Medicine (Application no. 5117). Thumbprint or signature consent from the mother, father or other head of the household was obtained after either parent read the consent form or it was explained to them by the fieldworker. This study was registered at clinicaltrials.gov as NCT00548379.

### Participants, randomisation and intervention

Infants aged 1–11 months living in five of 18 socio-economically deprived inner-city districts in Kabul were enrolled. A total of 3060 children were assessed for eligibility and 3,046 children were randomised by an independent statistician using unique identification numbers individually in fixed blocks of 20 to the placebo (*n* = 1522) or vitamin D3 (*n* = 1524) group by use of a random number generator with the SAS routine. The first dose of placebo or vitamin D was given by the fieldworker to the child at the recruitment visit. Placebo or vitamin D were administered at home, by trained staff, to the children on a quarterly basis (November 2007, February 2008, May 2008, August 2008, December 2008 and March 2009).

### Questionnaires and measurements

#### Baseline information

At recruitment, data on household socio-demographic characteristics and infant health were collected. Additional cross sectional data was gathered during follow-up. A wealth index was developed using principal component analysis based on household characteristics and assets. This index was divided into fifths as a final measure of the socio-economic status of a household.

#### Dietary intake of energy, protein and calcium

The parent or carer completed a semi-quantitative food frequency questionnaire at two time points through the study. Child feeding modality was categorized based on maternal report over 1 week prior to sampling as exclusive breastfeeding, mixed breastfeeding, or replacement feeding. The child’s food intake was estimated from a list of 56 commonly consumed foods over the past week.

In addition to total energy and protein intake, calcium intake was assessed since calcium deficiency (< 300 mg/day) can cause rickets independent of serum 25OHD concentrations [18]. The mean daily intake of energy (kcal), protein and calcium were calculated by multiplying the frequency of consumption by standard portion sizes and the calorie, protein and calcium content of the food or beverage, derived mainly from the *Food Composition Table for Afghanistan* (Food, Agriculture, Animal Husbandry and Information Management and Policy Unit. Food Composition Table for Afghanistan. Kabul: Nutrition Department, Ministry of Health, Afghanistan; SAARCFOODS; FAAHM Nutrition Unit, FAO, Kabul; 2004.) but supplemented with values from *McCance and Widdowson’s composition of foods integrated dataset* [19] where appropriate. For breastfed infants, an extra 506 kcal, 7.5 g of protein and 120 mg of calcium were added. Values were mainly derived from studies conducted in parts of the Gambia [20] due to lack of data for Afghan mothers. The average of the two dietary assessments were used to calculate the intake of kcal, protein, and calcium.

#### Anthropometry

Anthropometric measurements were performed, by trained staff, while the children were wearing light/no clothing and were collected at baseline and 18 months. The height was determined to the nearest 0.1 cm and the child’s weight was measured to within 10 g. Measurements were performed in duplicates and the average used in analysis. The anthropometric measurements were converted to standard deviation scores (SDS) using the WHO growth reference (WHO Anthro for personal computers, version 3.2.2, 2011: Software for assessing growth and development of the world's children. 2010) [21].

### Blood sampling and laboratory analysis

Blood samples from a randomly selected subset of 632 children (300 from the placebo and 332 from the vitamin D group) were collected for 25OHD analysis at five time points over the 18 months study period. The children were randomly selected for each round of blood sampling and the blood samples were collected at various times of the year to capture the pharmacodynamic and seasonal variation in blood concentrations of 25OHD.

Samples were stored at − 20 °C and analysed at the end of the study using the IDS-iSYS Multi-Discipline Automated Chemiluminescent assay (Immunodiagnostic Systems Ltd, Tyne and Wear, UK) at the Manchester Royal Infirmary, Manchester, UK (Supra-Regional Vitamin D Reference Laboratories accredited to ISO9001:2000 and ISO13485:2003 and participating in the Vitamin D Quality Assurance Scheme).

### Radiographs, Thacher scoring and automated radiogrammetry

Wrist and knee radiographs from a random subset of 641 children were obtained at study completion. Nutritional rickets can affect mineralisation and linear growth, radiographs were, therefore, examined for the presence of rickets using a validated rickets severity scoring system (Thacher score [22]). Scoring was undertaken by a Consultant Paediatric Radiologist. The Thacher scoring system reports the radiographic features of rickets such as the widening of the growth plate, degree of lucency and irregular margins at the metaphyses [22]. The wrist is scored for both the radius and the ulna and the knee for both the distal femur and proximal tibia. The severity of rickets is scored on a scale of 0 (normal) to 10 (severe) and a score of > 1.5 regarded as rickets [22]. After excluding over- or under-exposed radiographs (*n* = 10), Thacher scores were available on 631/641 sets of radiographs (310 in the placebo and 321 in the vitamin D group).

Good quality wrist radiograph films were available for 565 children for scanning, which was performed in groups of 25 using a film digitiser and transmitted to PACS (picture archiving and communication system) and subsequently burnt onto a CD. The images were DICOM (Digital Imaging and Communications in Medicine) files with 300dpi resolution. The images were then converted to JPEG images (average size of 800 kb) and analysed using BoneXpert version 3.1 to obtain automated BA and BHI. BHI is automated using the formula BHI = π × (1 − T/W)/ (LW)^0.33^. T is defined as the cortical thickness of the three middle metacarpals, W is the metacarpal width and L is the bone length.

All analysed images were visually reviewed for edge detection or other errors, and only the acceptable analyses were selected. Radiographs with reduced exposure were excluded (*n* = 43). Reference data from healthy Parisian children from 1955 [14] were used to compute BA SDS for age and the BHI SDS values for BA, as this is the only available source of validated reference data for children under 5 years of age. Since BA is a better indicator of the child’s physiological development than chronological age, BHI SDS is routinely computed using BA as a reference [14].

### Definition of terminologies

#### Malnutrition

In the context of the work described here, the terminology ‘malnutrition’ or ‘malnourished’ refers to ‘undernutrition’ or ‘undernourished’. In accordance with the WHO definition, undernutrition, includes wasting (low weight-for-height), stunting (low height-for-age) and underweight (low weight-for-age). Using the standard WHO reference [21], children with a z score or standard deviation score (SDS) of < − 2 for weight-for-height, height-for-age and weight-for-age were considered to have wasting, stunting and underweight, respectively.

#### Vitamin D deficiency

In accordance with the global consensus recommendations on prevention of rickets [18] and Institute of Medicine [23], 25OHD levels below 30 nmol/L were regarded as deficient, 30–50 nmol/L as insufficient and > 50 nmol/L as sufficient.

#### Skeletal maturity

Skeletal maturation was classed as “normal or advanced” if BA SDS was ≥ 0 and as “delayed” if BA SDS was < 0 to − 2 and severely delayed if BA SDS was < − 2.

### Statistical analysis

All statistical analyses were performed using Stata statistical software, V.15 (StataCorp, College Station, Texas, USA).

The difference in mean values of categorical variables was assessed using a chi-squared test and for continuous variables multiple linear regression used. Two-sided *p* values < 0.05 were considered statistically significant.

## Results

Of the 641 radiographs available, 565 good quality films were digitised. Nearly 93% (*n* = 522/565) of the available wrist images were analysable; 255 (138 males) in the placebo and 267 (153 males) in the vitamin D group. We report the findings from the cohort with a valid BA and BHI (*n* = 522).

### General characteristics

The mean (± SD) age of the cohort was 2 (± 0.3) years. The general characteristics for the study population are presented in Table 1. The placebo and intervention (vitamin D) groups were similar in terms of age and gender. The two groups had comparable dietary intake, including calcium, and socio-economic status.

**Table 1:** Characteristics of placebo and Vitamin D groups with anthropometric data at study completion

Characteristics	All	Placebo	Vitamin D
*N*	522	255	267
Age in years (SD)	2.0 (0.3)	2.0 (0.3)	2 (0.3)
Gender: number of girls (percentage)	231 (44.3%)	117 (45.9%)	114 (42.7%)
Height-for-age SDS (SD)	− 2.2 (1.1)	− 2.1 (1.1)	− 2.2 (1.0)
Weight-for-age SDS (SD)	− 1.3 (1.0)	− 1.3 (1.0)	− 1.4 (0.9)
Weight-for-height SDS (SD)	− 0.3 (1.0)	− 0.3 (1.0)	− 0.3 (1.0)
Mean height velocity in cm/ year (SD)	10.7 (3.0)	10.8 (3.1)	10.5 (3.0)
Mean total energy intake in kcal (SD)	740.2 (331.2)	730.8 (335.0)	749.5 (327.8)
Mean protein intake in g/d (SD)	21.9 (10.2)	21.7 (10.1)	22.0 (10.3)
Mean calcium intake in mg/d (SD)	382.4 (286.2)	398.1 (287.5)	366.9 (284.7)
Mean serum 25OHD 3 months after treatment end in the summer, in nmol/L (95% CI)		47 (95% CI 41, 56)	55 (95% CI 45, 57)
Thacher score number (percentage)			
< 1	373 (72.9%)	174 (70.5%)	199 (75.1%)
≥ 1 to ≤ 1.5	115 (22.5%)	61 (24.7%)	54 (20.4%)
> 1.5	24 (4.7%)	12 (4.9%)	12 (4.5%)
Socio-economic group number (percentage)			
Better off	62 (13.7%)	30 (13.7%)	32 (13.7%)
Less poor	107 (23.6%)	49 (22.4%)	58 (24.8%)
Poor	92 (20.3%)	49 (22.4%)	43 (18.4%)
Very poor	79 (17.4%)	38 (17.4%)	41 (17.5%)
Poorest	113 (24.9%)	53 (24.2%)	60 (25.6%)

**p < 0.001, *p < 0.05

### Prevalence of malnutrition

At study completion, in the cohort with analysable radiographs (*n* = 522) the prevalence of stunting (height for age SDS < − 2 SD), underweight (weight for age SDS < − 2 SD) and wasting (weight for height SDS < − 2 SD) were 54.7% (*n* = 233), 24.6% (*n* = 105) and 4.7% (*n* = 20), respectively. The total daily calorie intake in the study cohort [mean (SD) 740.2 (331.2); *n* = 522] was lower than expected for an average healthy 24-month-old (mean total daily calorie intake of 740 vs. 1000 kcal).

### Bone age and Bone Health Index

**Fig. 1:**
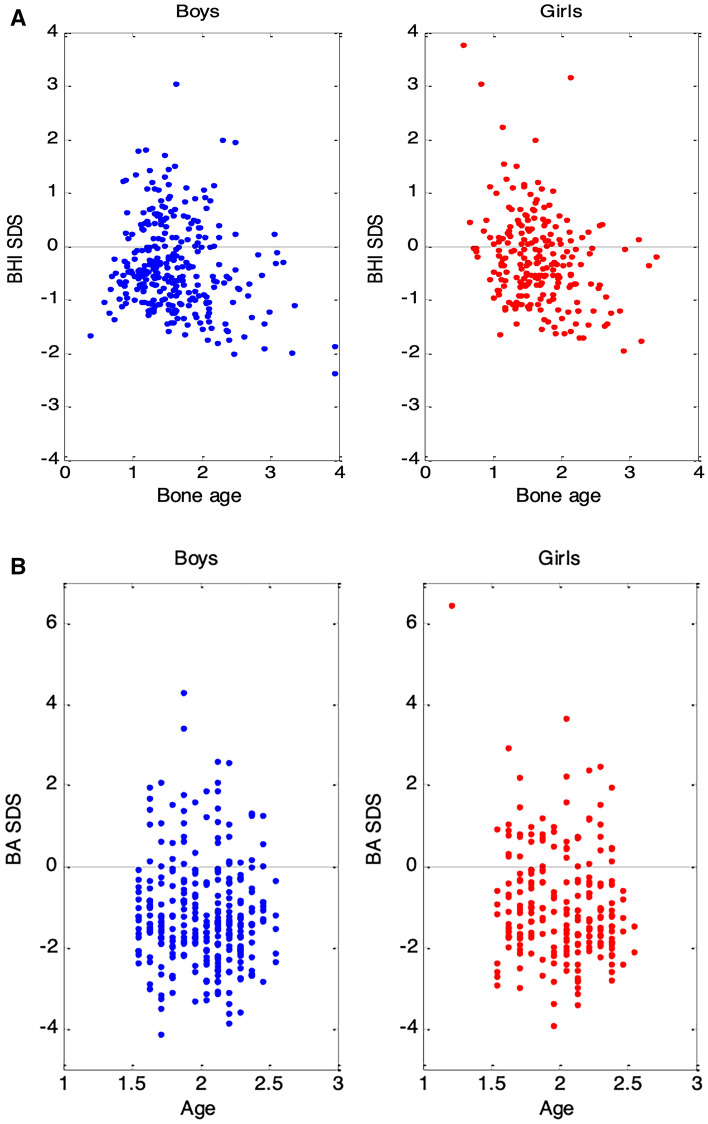
**a** Mean bone age is lower compared to mean chronological age in both boys and girls, **b** mean bone age standard deviation score (BA SDS) was lower in boys compared to girls

BA was delayed compared to chronological age in both boys [mean (± SD) 1.57 (0.50) vs 2.00 (0.30) years, respectively] and girls [mean (± SD) 1.68 (0.52) vs 2.00 (0.30), respectively], Fig. 1a. BA SDS was significantly lower than zero in the whole study group [mean (95% CI) -1.07 (95% CI − 1.19 to − 0.96, *p* < 0.001)]. BA SDS was significantly lower in boys than in girls [mean (± SD) − 1.22 (1.25) and − 0.89 (1.42), respectively; difference (95% CI) of 0.33 SDS (0.10 to 0.56); *p* < 0.01], Fig. 1b.

BHI SDS was statistically significantly different from zero [mean (95% CI) − 0.31 (95% CI − 0.38 to − 0.24, *p* < 0.001)], with a tendency for lower values in boys compared to girls [mean (± SD) − 0.35 (0.82) and − 0.26 (0.86), respectively; difference (95% CI) of 0.08 SDS (− 0.06 to 0.23)].

### Effect of vitamin D supplementation on 25OHD, BA and BHI

The mean serum 25OHD concentration in the placebo group was significantly lower than in the intervention group; when measured 1 month after the first dose in autumn (November–December 2007) [mean (95% CI) 39 nmol/L (95% CI 35–44) vs. 115 nmol/L (95% CI 103–128), *p* < 0.001). Four months after treatment end, in summer months (July–August 2009) at the time of radiographs, there was no difference in mean 25OHD concentrations between the placebo and vitamin D groups [mean (95% CI) 47 nmol/L (95% CI 41–54) vs 51 nmol/L (95% CI 45–57), *p* = 0.2]. The placebo vs vitamin D groups had similar BA SDS [mean (± SD) -1.04 (1.36) vs. − 1.14 (1.26)]. There was no statistically significant difference in BA SDS between the adequate (≥ 300 mg/day) vs inadequate (< 300 mg/day) calcium intake groups (Table 2). The BA SDS and BHI SDS for various categorical variables are presented in Table 2.

**Table 2:** Bone age SDS and Bone Health Index SDS for various categorical variables at study completion

	Bone age SDS (SD)	Bone Health Index SDS (SD)
Group		
Placebo	− 1.04 (1.36)	− 0.30 (0.86)
Vitamin D	− 1.14 (1.26)	− 0.31 (0.80)
Difference (95% CI)	− 0.11 (− 0.34 to 0.12)	− 0.01 (− 0.15 to 0.14)
Sex		
Boys	− 1.22 (1.25)	− 0.35 (0.82)
Girls	− 0.89 (1.42)	− 0.26 (0.86)
Difference (95% CI)	0.33 (0.10–0.56)**	0.08 (− 0.06 to 0.23)
Socio-economic group		
Better off	− 0.87 (1.28) − 0.87 (1.28)	− 0.29 (0.73) − 0.29 (0.73)
Less poor	− 0.97 (1.39)	− 0.24 (0.92) − 0.24 (0.92)
Poor	− 1.01 (1.36)	− 0.37 (0.90)
Very poor	− 1.25 (1.28)	− 0.42 (0.84) − 0.42 (0.84)
Poorest	− 1.32 (1.12) − 1.32 (1.12)	− 0.27 (0.82)
*P* for trend	0.099	0.595
Thacher score		
< 1	− 1.01 (1.32)	− 0.29 (0.86)
1–1.5	− 1.16 (1.42)	− 0.29 (0.72)
> 1.5	− 1.68 (0.98)	− 0.44 (0.84)
*P* for trend	0.042*	0.702
Calcium intake		
< 300 mg/d	− 1.17 (1.29)	− 0.35 (0.81)
≥ 300 mg/d	− 1.07 (1.22)	− 0.25 (0.88)
Difference (95% CI)	0.10 (− 0.14 to 0.34)	0.11 (− 0.05 to 0.26)

**p < 0.001, *p < 0.05

Bone age delay was significantly greater in children with rickets (Thacher score > 1.5) than in those without rickets [mean BA SDS (± SD) − 1.68 (0.98) and − 1.04 (1.35), respectively; Difference (95% CI) of − 0.64 (− 1.18 to − 0.09), *p* < 0.05]. The mean BHI SDS (± SD) did not differ between those with and without rickets (Table 2).

### Nutritional and anthropometric differences in children with advanced vs delayed bone age

**Fig. 2:**
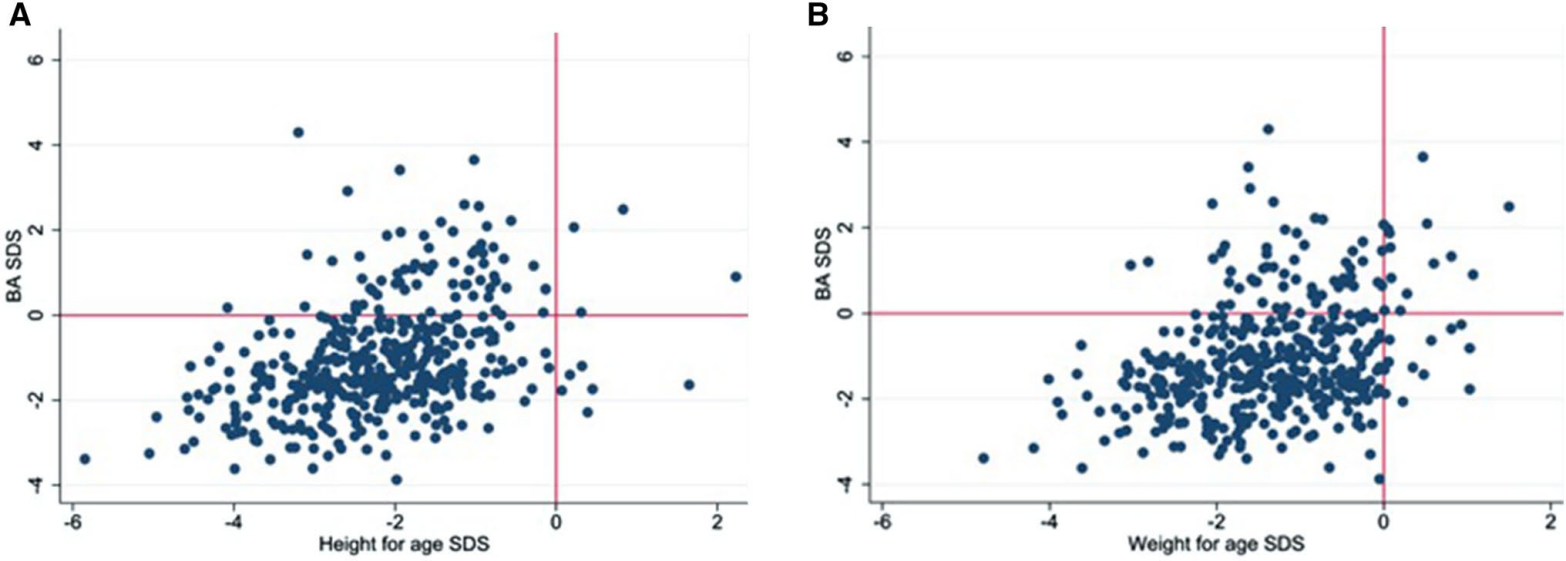
Children with a delayed bone age (BA SDS < 0) were significantly more stunted (**a**) and underweight (**b**) than children with a normal bone age (BA SDS > 0)

The nutritional intake and anthropometry of children with advanced BA when compared to those with delayed BA are presented in Table 3. Children with advanced BA had consumed more total calories than those with delayed BA [mean total calories (± SD) 805 (± 346) vs 723 (± 327) kcal/day, respectively, *p* < 0.05], were significantly less stunted (Fig. 2a) [height-for-age SDS (± SD) of − 1.43 (± 0.9) vs. − 2.3 (± 1.0), respectively, *p* < 0.001] and underweight (Fig. 2b) [weight-for-age SDS (± SD) of − 0.82 (± 0.8) vs. − 1.45 (± 0.9), respectively, *p* < 0.001]; and had a greater growth velocity [mean annual height velocity (± SD) of 11.57 (± 2.7) vs. 10.47 (± 3.0) cm/year, respectively, *p* < 0.05]. The odds of stunting for each one-unit increment in bone age SDS was 0.54 (95% CI 0.45–0.65), *p* < 0.001. There was no difference in protein or calcium intake between the two groups (Table 3). The nutritional intake and anthropometry of children with delayed skeletal maturity compared to those with severely delayed skeletal maturity are presented in Table 4.

**Table 3:** Differences in characteristics of children with advanced skeletal maturity (BA SDS ≥ 0) and delayed skeletal maturity (BA SDS < 0)

Characteristics	BA SDS ≥ 0	BA SDS < 0	*p* value
*N*	91	421	
Age (years)	2.0 (0.25)	2.0 (0.26)	0.251
Height-for-age SDS (SD)	− 1.43 (0.9)	− 2.32 (1.0)	< 0.001
Weight-for-age SDS (SD)	− 0.82 (0.8)	− 1.45 (0.9)	< 0.001
Weight-for-height SDS (SD)	− 0.12 (0.9)	− 0.31 (1.0)	0.126
Mean height velocity in cm per year (SD)	11.57 (2.7)	10.47 (3.0)	< 0.05
Mean total energy intake in kcal (SD)	805 (346)	723 (327)	< 0.05
Mean protein intake in g/day (SD)	23 (11)	21(10)	0.26
Mean calcium intake in mg/day (SD)	429 (330)	370 (275)	0.107

**Table 4:** Differences in characteristics of children with delayed skeletal maturity (BA SDS − 2 to < 0) and severely delayed skeletal maturity (BA SDS < − 2)

Characteristics	BA SDS − 2 to < 0	BA SDS < − 2	*p* value
*N*	308	113	
Age (years)	2.0 (0.27)	2.0 (0.25)	0.18
Height-for-age SDS (SD)	− 2.15 (0.9)	− 2.80 (1.0)	< 0.001
Weight-for-age SDS (SD)	− 1.33 (0.9)	− 1.77 (1.0)	< 0.001
Weight-for-height SDS (SD)	− 0.28 (1.0)	− 0.40 (0.9)	< 0.001
Mean height velocity in cm per year (SD)	10.6 (3.0)	10.1 (3.0)	0.28
Mean total energy intake in kcal (SD)	733 (333)	695 (310)	0.33
Mean protein intake in g/day (SD)	21 (10)	22 (10)	0.66
Mean calcium intake in mg/day (SD)	370 (272)	368 (286)	0.96

The nutritional and anthropometric differences between children with BHI greater than or lower than baseline (zero) are provided in the Supplementary Table 1.

## Discussion

This study shows that deprived Afghan children, boys in particular, had a lower mean BA and BHI compared to zero (reference population). Children with delayed BA were significantly more stunted and underweight than children with advanced BA but both groups had adequate bone density as assessed by BHI. Skeletal maturity was significantly influenced by the total calorie consumption, but not protein and calcium intake or bolus vitamin D supplementation.

### Malnutrition

In comparison to the 2013 National Nutrition survey [4], our cohort had a higher prevalence of stunting (40.9% vs. 54.7%) and lower prevalence of wasting (9.5% vs 4.7%). We suspect that the differences may be due to the variation in study period and the age groups studied. In addition, our cohort was from a socio-economically deprived region as opposed to the nationally representative sample included in the National Nutrition survey.

### Effect of vitamin D supplementation

In our population, quarterly vitamin D supplementation did not influence BA, BHI or height velocity. Nutritional rickets resulting from vitamin D and/or calcium deficiency [24] is frequently encountered in malnourished children [25]. Poor mineralisation of the growth plates in nutritional rickets results in poor linear growth, stunting [26] and delayed bone age. A study in Ecuadorian children aged 6–36 months (*n* = 516) reported that serum 25OHD levels below 42.5 nmol/L were associated with stunting and underweight [6]. A randomised controlled trial from New Delhi, India reported a positive effect on growth and reduction of stunting in low birth weight term infants (*n* = 2079) who received weekly vitamin D supplementation (at 35 μg/week) for 6 months [27]. Therefore, potential reasons for the lack of response in BA, BHI or height velocity to supplementation in our cohort include the lack of vitamin D deficiency at baseline, the frequency of bolus doses and the possibility of BA delay being multifactorial. Serum 25OHD concentration 1 month after the first bolus dose in late autumn (regarded as baseline) in the placebo group was in the insufficiency range (30–50 nmol/L) [18]. We know from systematic reviews on the prevention of respiratory infections that benefits of vitamin D supplementation are maximal in children with lower baseline 25OHD (< 25 nmol/L) and in those who receive daily or weekly supplementation as opposed to bolus doses [28]. Similar to stunting, BA delay in malnourished children is also likely to be multifactorial and include nutritional and non-nutritional factors [29, 30].

Presence of rickets resulted in a significant BA delay with higher Thacher severity score; however, the BHI did not differ between children with and without rickets. BHI merely reports bone geometry (incorporating cortical thickness, width and length) [13] and does not distinguish between mineralised or poorly-mineralised cortex thereby limiting its utility in the diagnosis of rickets.

### Nutritional and anthropometric differences in children with advanced vs. delayed bone age

Children with BA delay were significantly more stunted and had a lower height velocity than children with advanced BA. Although stunting can be secondary to a multitude of factors including maternal attributes such as height, nutrition and education [29], the presence of lower total calorie consumption and BA delay in our stunted cohort indicates nutritional growth retardation [31]. Skeletal maturity was influenced by total calorie intake rather than protein or calcium intake in our cohort. A previous study in rural Guatemalan malnourished children evaluated the effect of two different types of dietary supplements; a combined high calorie dietary supplement with protein, mineral and vitamin A (average SD daily calorie of 94 ± 76 kcal) vs a low calorie dietary supplement without protein (average SD daily calorie of 16 ± 13 kcal) [15]. The study found that the higher the intensity of intervention (total calories, proteins and vitamins) the better the response in growth; however, they were not able to evaluate the effect of individual nutrients due to the combined high calorie dietary supplement used [15]. The study also found that the effect of dietary intervention on body size (height and weight) was more pronounced than the effect on skeletal maturity as determined by the number of ossification centres [15]. A study in Chilean survivors of protein energy malnutrition found that rehabilitated children remained shorter than the control healthy group despite a catch up in bone age; indicating the role of genetic or prenatal/maternal factors in stunting [32].

Children with severe BA delay were more malnourished (stunted, wasted and underweight), but had a similar total calorie intake to those with delayed BA indicating the role of other potential contributors in this scenario. In addition to factors such as health status, dietary in-take and food availability, stunting in Afghan children is also influenced by maternal health, health environment and services, and public policies and law [30]. Socio-economic status plays an important role in maternal and child health. We observed a clear downward trend in skeletal maturity in children from lower socio-economic strata; however, this did not reach statistical significance. A similar positive association between nutritional status and wealth index quintiles was reported in the Afghan National Nutrition Survey [4].

Boys had a significantly lower mean BA SDS when compared to girls. The National Nutrition Survey reports a significantly higher (*p* < 0.001) prevalence of stunting in males (42.3%; 95% CI 40.5–44.1) compared to females (39.4%; 95% CI 37.5–41.3) [4]. The higher rates of under-nutrition among boys compared to girls has been attributed to factors such as higher morbidity among boys, a higher exclusive breastfeeding rate and better dietary diversity among girls [4].

### BHI

Contrary to bone age, the BHI showed minimal variations between gender or height because BHI is calculated for a given bone age [13], rather than chronological age. We therefore postulate that the bone health, determined using BHI, for the given skeletal age in these children is satisfactory and not influenced by vitamin D supplementation.

Stunting caused by undernourishment is associated with impaired brain development which leads to lower cognitive and socioemotional skills, lower levels of educational attainment, and hence lower incomes; ultimately resulting in a lower per capita income [33]. In 2015, the Afghanistan’s Government signed the Kabul Declaration, renewing its commitment to reduce preventable deaths among women and children by 2020: ‘A Promise Renewed’[30]. The Government is also committed to the Global strategy for Women’s, Children’s and Adolescent’s health (2015–2030) and a key target is reduction in the rate of stunting in children under 5 years of age to 30% by 2020 and 10% by 2030 [30]. As a result of these commitments the Ministry of Health has considered a number of nutrition-specific and nutrition-sensitive initiatives [30] focusing on the first 1000 days of life [34]. The radiogrammetry findings presented here precede the nutritional initiatives in Afghanistan, and therefore, provide benchmarking data against which the impact of the Government’s ongoing nutritional initiatives can be assessed in the future.

The study was limited by the lack of radiographs at baseline and follow up to compare individual response to supplementation, nonetheless we were able to evaluate response against the placebo group. Another potential limitation is that the subgroups were selected at random for both biochemical and radiological evaluation with little overlap between the two groups.

## Conclusion

Malnourished children, in particular boys, have significant delay in skeletal maturation, but no substantial impairment in bone density as assessed by BHI. Quarterly oral vitamin D supplementation did not influence BA delay but total calorie intake did; highlighting the importance of overall calorie intake. Dietary interventions for malnutrition should place more emphasis on total calories as children with more calorie consumption had better height velocity and improved skeletal maturity. Future studies evaluating the effect of vitamin D supplementation on growth and skeletal maturity should consider daily or weekly supplements. The first report of BA in the malnourished population can serve as user independent benchmark data against which the impact of the health and nutrition initiatives in low- and middle-income countries, and specifically Afghanistan, can be assessed.

## Supplementary Information

Below is the link to the electronic supplementary material.

Supplementary file1 (DOCX 109 KB)
