# Supplementary material for: The effect of vitamin D supplementation and nutritional intake on skeletal maturity and bone health in socio-economically deprived children
Source: Eur J Nutr. 2021 Feb 20;60(6):3343–53. doi: 10.1007/s00394-021-02511-5 (PMC8354903; doi:10.1007/s00394-021-02511-5)
Supplement: Supplementary file 1 [file 394_2021_2511_MOESM1_ESM.docx]

Supplementary table 1: Differences in characteristics of children with Bone Health Index gre than zero.

Characteristics	BHI < 0	BHI ≥ 0
n	359	163
Age (months)	24.7 (3.2)	24.0 (3.0)
Height-for-age z score	-2.1 (1.1)	-2.3 (1.0)
Weight-for-age z score	-1.3 (1.0)	-1.5 (1.0)
Weight-for-height z score	-0.2 (1.0)	-0.4 (1.0)
Height velocity (cm per year)	10.6 (3.0)	10.9 (3.2)
